# Applying MCM-48 mesoporous material, equilibrium, isotherm, and mechanism for the effective adsorption of 4-nitroaniline from wastewater

**DOI:** 10.1038/s41598-023-37090-4

**Published:** 2023-06-17

**Authors:** Nisreen S. Ali, Hamed N. Harharah, Issam K. Salih, Noori M. Cata Saady, Sohrab Zendehboudi, Talib M. Albayati

**Affiliations:** 1 Materials Engineering Department, College of Engineering, Mustansiriyah University, Baghdad, Iraq; 2 Department of Chemical Engineering, College of Engineering, King Khalid University, 61411 Abha, Kingdom of Saudi Arabia; 3 Department of Chemical Engineering and Petroleum Industries, Al-Mustaqbal University College, Babylon, 51001 Iraq; 4 Department of Civil Engineering, Memorial University of Newfoundland, St. John’s, NL A1B 3X5 Canada; 5 Department of Process Engineering, Memorial University of Newfoundland, St. John’s, NL A1B 3X5 Canada; 6 Department of Chemical Engineering, University of Technology-Iraq, 52 Alsinaa St., P.O. Box 35010, Baghdad, Iraq

**Keywords:** Environmental chemistry, Engineering, Chemical engineering

## Abstract

In this work, the MCM-48 mesoporous material was prepared and characterized to apply it as an active adsorbent for the adsorption of 4-nitroaniline (4-Nitrobenzenamine) from wastewater. The MCM-48 characterizations were specified by implementing various techniques such as; scanning electron microscopy (SEM), Energy dispersive X-ray analysis (EDAX), X-ray diffraction (XRD), Brunauer–Emmett–Teller (BET) surface area, pore size distribution (PSD), and Fourier transform infrared (FTIR). The batch adsorption results showed that the MCM-48 was very active for the 4-nitroaniline adsorption from wastewater. The adsorption equilibrium results were analyzed by applying isotherms like Langmuir, Freundlich, and Temkin. The maximum experimental uptake according to type I Langmuir adsorption was found to be 90 mg g^−1^ approximately. The Langmuir model with determination coefficient R^2^ = 0.9965 is superior than the Freundlich model R^2^ = 0.99628 and Temkin model R^2^ = 0.9834. The kinetic adsorption was investigated according to pseudo 1st order, pseudo 2nd order, and Intraparticle diffusion model. The kinetic results demonstrated that the regression coefficients are so high R^2^ = 0.9949, that mean the pseudo 2nd order hypothesis for the adsorption mechanism process appears to be well-supported. The findings of adsorption isotherms and kinetics studies indicate the adsorption mechanism is a chemisorption and physical adsorption process.

## Introduction

One of the biggest environmental problems in the world is water contamination caused by chemical wastes, particularly in developing nations. Aromatic substances, such as 4-nitroaniline (4-NA), are one of the typical contaminants in industrial wastewater from the production of insecticides, petrochemicals, chemical paints, and oil refineries^1–3^. This substance causes significant environmental issues because of its toxicity, mutagenicity, and carcinogenicity^4^. The US Environmental Protection Agency (USEPA) has identified this substance as one of the most significant and urgent water contaminants. Even at extremely low concentrations, 4-NA in water poses a threat to human health and aquatic life^5–7^. Different treatment methods, such as membrane processes, coagulation, biodegradation, and adsorption, have been developed to treat industrial wastewater. Still, their use is constrained by issues like high expenses, ineffectiveness, and noncompliance with discharge effluent regulations^8–10^. Furthermore, a few of these processes just condense persistent organic pollutants from the liquid phase (water) into the solid phase by condensation. As a result, they necessitate additional expenditures for the treatment of secondary contaminants^11–14^. Adsorption techniques have been applied to eliminate inorganic and organic pollutants from wastewater, with a particular emphasis on the usage of various materials as the preferred adsorbent. It may be said that the regeneration of the used adsorbent materials is a time-consuming and expensive procedure^15^. Because of this, there is interest in creating new adsorbents to remove contamination in the aqueous waste stream^16^. Based on the size, shape, and other characteristics of a molecule, such as a polarity, Zeolites can reject or selectively adsorb certain compounds. This means that they can act as adsorbents. Organ clays have been the focus of several investigations on organic molecule adsorption from aqueous solutions. There have been reports on silicate^16^, mesoporous materials^17^, and modified and unmodified zeolites^18–20^. For application in separation procedures as an adsorbent, the candidate MCM-48 appears to be more promising. Scientists have been very interested in the mesoporous material MCM-48 since it was discovered by Mobil Oil researchers in 1992 because of its potential use as support of catalysts, catalysts, and absorbents. These material properties are high thermal stability, specific pore volume up to 1.2 cm^3^ g^−1^, surface areas (1000–1500 m^2^ g^−1^), a narrow pore-size distribution, and ‘‘non-cytotoxic’’ properties^21^. Mesoporous silica-based materials, including MCM-48, have been developed as efficient catalyst carriers, adsorbents, and cutting-edge drug delivery systems^22^. This is due to their very considerable thermal stabilities, porous morphologies, huge surface areas, and highly reactive surfaces for the presence of the silanol groups^23,24^.

In this work, the mesoporous materials MCM-48 were investigated as an efficient adsorbent for the removal of (C_6_H_6_N_2_O_2_) dye from industrial wastewater in a batch adsorption process. The adsorption isotherms and kinetics were examined. Moreover, the adsorption mechanism of 4-nitroaniline sewage onto the surface of MCM-48 adsorbent was studied in a batch adsorption process. Hereon, it is described how the surface characteristics of the adsorbent are critical to determining the adsorption properties of 4-nitroaniline (4-nitrobenzenamine) dye. Finally, regeneration and desorption kinetics were also tested to discover the actual adsorbent utility and its applicability for reusability. Mesoporous materials MCM-48 have already been used for the elimination of many kinds of pollutants from wastewater. To our knowledge, very few publishing results are recommended on the investigation of 4-nitroaniline (4-nitrobenzenamine) removal from aqueous solution by MCM-48 adsorbent. It is gaining particular attention and is viewed as a possible substitute for traditional water treatment in the textile sectors.

## Experimental

### Chemicals

The chemicals applied for this work were cetyl trimethyl ammonium bromide C_19_H_42_BrN (CTAB; purity > 98%) as a surfactant, tetraethyl orthosilicate Si(OC_2_H_5_)_4_ (TEOS; purity > 98% (as a silica source, sodium hydroxide (NaOH), hydrochloric acid (HCl), and 4-nitroaniline (4-nitrobenzenamine) (C_6_H_6_N_2_O_2_). All reactants were analytically purchased from Sigma Aldrich Chemical Company. All materials were applied without additional purification.

### Preparation of MCM-48

According to the preparation method outlined by^25–27^, MCM–48 was synthesized. The following steps were used to create MCM-48 in a representative preparation: 90 g of deionized water mixed with 10 g of CTAB. 1 g of NaOH was then mixed with the solution after it had been rapidly agitated at 35 °C for 40 min. Eleven cm^3^ of TEOS was added, after stirring the solution for 60 min at 35 °C, and the mixture was then stirred at this temperature for another 30 min. Final heating of the combination was place in an autoclave under static conditions for 24 h at 150 °C; the resultant MCM-48 was then cooled down for 1 h, filtrated, and rinsed with distilled H_2_O before being dry at ambient temperature. The produced sample was then calcined for 6 h at a temperature of 650 °C applying a ramp rate for heating 2 °C/min.

### Characterization

The MiniFlex (Rigaku) diffractometer was used to record the patterns of small-angle XRD in ambient settings using Cu K radiation (λ = 1.5406 Å). The X-ray tube was run at 40 kV and 30 mA, and the data were reported in the 2θ range of 0.5–8° with a 2 step size of 0.01 and a step time of 10 s. The formulae nλ = 2dsinθ and ɑ_o_ = 2d100/$\surd$ 3 were applied to determine the unit cell and d-spacing characteristics. The pore analyzer of a micrometrics ASAP 2020 was used to assess the adsorption and desorption of nitrogen using N_2_ physisorption at   196 °C. All specimens were degassed in the degas adsorption analyzer port for 3 h at 350 °C and vacuum (*p* < 10^–5^ mbar). The BET method was used to calculate the specimens' BET-specific surface areas for the relative pressure range of 0.05–0.25. With the use of the Barrett-Joyner-Halenda (BJH) approach, which is based on thermodynamics, the distributions of pore size were specified from the isotherm desorption branch. The quantity of liquid N_2_ adsorbed at P/P_0_ = 0.995 was used to calculate the total pore volume. This information was obtained from the N_2_ isotherm's adsorption branch. Through the use of the unit cell parameter(ɑ_o_) and pore size diameter, the pore walls thickness (t_W_) was estimated (d_P_). Using BET analysis (4 V/A), the average mesopore sizes for the single specimens were calculated from the data of nitrogen sorption. SEM was carried out on a JEOL (JSM-5600 LV). EDAX is an analytical method that creates the adsorbent elemental analysis to determine the composition of chemicals when combined with SEM. Using a NICOLET 380 FT-IR spectrometer, the solid samples' infrared spectra were measured were in the 4000 to 400 cm^−1^ range at areas with 4 cm^−1^ resolutions in transmission mode at ambient temperature.

### Experiments of batch adsorption

To assess 4-nitroaniline isotherms of adsorption onto the adsorbents at 25 °C, batch adsorption tests were performed. 4-Nitroaniline stock solutions were created by dissolving 0.2 g in 1 L of distilled H_2_O. At 25 °C, experiments of batch adsorption were used to assess the 4-nitroaniline adsorption over the adsorbents. By dissolving 0.2 g of 4-nitroaniline in 1 L of distilled water, stock solutions of 3-Nitroaniline were created. Then, 10 concentrations (0–0.2 g/L) were used to create a calibration curve using a UV-Spectrometer (model HP 8453) calibrated to 25^0^C. λmax was discovered to be 278 nm. The calibration was necessary to compare final absorbance with beginning absorbance. In 100 ml conical flasks, 15 different concentrations of the aforementioned solutions were created, starting from 0.001 to 0.06 g/L. 100 ml of each was added to 0.01 g MCM-48, which was then stirred in several positions at 150 rpm for an hour at room temperature (25 °C). This made it possible for the mesoporous substance MCM-48 to completely mix with the mixture. Following the adsorption procedure, equal quantities of the solutions were centrifuged for 5 min at 3500 rpm using a centrifuge (model Hermle Z 200 A). This allowed the zeolite to completely separate from the solution and enable analysis with a UV-spectrophotometer (TU1900) operating at a wavelength of 278 nm. According to the following equation, the (%R) of 4-nitroaniline was calculated^28^:

1 $$\%R=\frac{\mathrm{Co}-\mathrm{Ce}}{\mathrm{Co}}\times 100\%$$

Using the following equation, the amount of adsorption (qe) was determined ^29^:

2 $$qe=\frac{\mathrm{V}(\mathrm{Co}-\mathrm{Ce})}{\mathrm{m}}$$

where, $qe$(mg / g) denotes the amount of adsorption, V (L) is the 4-nitroaniline solution volume, m (g) is the absorbent mass applied in the experiments, $\mathrm{Ce}$ (mg/L) and C_0_ (mg/L) are the equilibrium and initial concentration of 4-nitroaniline, respectively. Where qe (mg/g) is the amount of 4-nitroaniline that will be absorbed, V (L) is the volume of the 4-nitroaniline solution, C_0_ (mg/L) and Ce (mg/L) are the 4-nitroaniline starting and equilibrium concentrations in the liquid phase, and m (g) is the absorbent mass that will be utilized in the experiment. The metal ions uptake at any time was calculated from Eq. (2).

3 $${q}_{t}=\frac{\mathrm{V}({C}_{o-}{C}_{t})}{\mathrm{W}}$$

where: ${q}_{t}$ and ${C}_{t}$ are the adsorption capacity in (mg/g) and 4-nitroaniline concentration in solution in (mg/L) respectively at time t^30^.

### Adsorption isotherms models

The 4-nitroaniline removal from an aqueous solution was studied applying the adsorption isotherm under equilibrium circumstances to determine the connection between the amount of 4-nitroaniline adsorbed on the MCM-48 and that which is left in the aqueous solution. The experimental data from the investigation was fitted to three distinct Langmuir, Freundlich, and Temkin isotherm models.

#### Langmuir isotherm model

The model of Langmuir adsorption is based on the concept of monolayer adsorption, according to which adsorption can only take place at fixed-number, equivalent, and identical spatially separate locations. The Langmuir equilibrium adsorption equation has the following form^31^.

4 $${q}_{e}=\frac{{q}_{max}b{C}_{e}}{1+b{C}_{e}}$$

where: ${q}_{e}$ is the adsorbent's equilibrium adsorption capability in mg/g. b is the constant of Langmuir in (L/mg), which measures the degree of the adsorbate's tendency for adsorption. A high value of b implies significantly better affinity of metal ion adsorption. ${q}_{max}$ is the maximum capacity for adsorption in (mg/g), ${C}_{e}$ is the concentration of equilibrium in (mg/L), and b is the Langmuir constant in (L/mg). Equation (4) has the following linear form:

5 $$\frac{{C_{e} }}{{q_{e} }} = \frac{1}{{ q_{\max } }}C_{e} + \frac{1}{{ bq_{\max } }}$$

The equilibrium parameter (RL), often known as the separation factor or dimensionless constant, may be written as^32^:

6 $$R_{L} = \frac{1}{{1 + bC_{o} }}$$

This parameter shows the isotherm is unfavorable (RL > 1), favorable (R_L_ < 1), linear (R_L_ = 1), or irreversible (R_L_ = 0)^33^.

#### Freundlich isotherm model

The first equation to represent reversible, non-ideal adsorption that is not predicated on monolayer formation is the Freundlich model. An empirical equation called the Freundlich isotherm is used to describe a heterogeneous system, it is stated as:

7 $${q}_{e}={K}_{f}{C}_{e}^{1/n} n>1$$

The equation for the linearized Freundlich isotherm is:

8 $$Log qe=Log KF+\left(\frac{1}{n}\right)Log Ce$$

where; ${K}_{f}$ and $n$ are Freundlich constants related to capacity of adsorption and intensity of adsorption, respectively. The slope and intercept of the linear plot for experimental data $log$ ${q}_{e}$ versus $log$ ${\mathrm{C}}_{\mathrm{e}}$ might be used to derive the values of n and ${K}_{f}$. Where the intercept = $log{K}_{f}$, and the slope = (1/n). The slope, which ranges from zero to unity, indicates the strength of adsorption or the heterogeneity of the surface since it is so low (near zero). While a slope close to one indicates chemisorption, a slope greater than one indicates cooperative adsorption^34^.

#### Temkin isotherm model

According to the model of Temkin isotherm, adsorption is characterized by a uniform distribution of binding energies up to a maximum binding energy, and all molecules’ adsorption heat reduces linearly with an increase in the amount of the adsorbent surface covered. The expression for this model is as follows^35^:

9 $${q}_{e}=\frac{\mathrm{RT}}{{b}_{T}}ln{K}_{T}{C}_{e}$$

where; ${K}_{T}$ is the equilibrium binding constant (L/g), which is the same as the extreme binding energy, ${b}_{T}$ is the Temkin isotherm constant, which refers to adsorption heat (8.314 J/mol K), and T is the absolute temperature (K). Equation (9) can be expressed in the linear form as follows:

10 $${q}_{e}=\frac{RT}{{b}_{T}}ln{K}_{T}+ \frac{RT}{{b}_{T}}ln{C}_{e}$$

A plot of ${q}_{e}$ versus $\mathrm{ln}$ ${\mathrm{C}}_{\mathrm{e}}$ enables the determination of the isotherm constants ${K}_{T}$ and ${b}_{T}$.

The isotherm constants ${K}_{T}$ and ${b}_{T}$ can be determined using a plot of ${q}_{e}$ versus $\mathrm{ln}$ ${\mathrm{C}}_{\mathrm{e}}$.

### Adsorption kinetics

In order to understand the phase of rate-limiting in the mechanism of adsorption in a particular system, contact time from experimental findings might be employed. The most often used models for studying the materials adsorption kinetics onto adsorbent are the pseudo-first-order, pseudo-second-order, and intraparticle models.

#### Pseudo first-order model

Pseudo-first-order kinetic equation can be written as follows^36^:

11 $$\frac{d{q}_{t}}{dt}={k}_{p1}\left({q}_{e}-{q}_{t}\right)$$

where;${q}_{e}$ and ${q}_{t}$ are the amount adsorbed (mg/g) at equilibrium and at time t, respectively. Integration and rearrangement of Eq. (11) give:

12 $$Log \left(qe-qt\right)=Logqe-{k}_{1}t$$

where; ${k}_{1}$ is the adsorption rate constant (h^−1^) for the pseudo first-order and can be determined from the linear plot's slope of log (*q*_e_ − *q*_t_) versus *t*.

#### Pseudo second-order model

The pseudo second order kinetic equation is given as^37^:

13 $$\frac{d{q}_{t}}{dt}={k}_{p2}{({q}_{e}-{q}_{t})}^{2}$$

Typically, the differential equation is integrated and converted to linear form:

14 $$\frac{t}{\mathrm{qt}}=\frac{1}{{k}_{2}{qe}^{2}}+\frac{1}{qe}t$$

where; ${k}_{2}$ is the adsorption rate constant (g/mg.min) for the pseudo second-order and can be calculated by plotting *t*/*q*_t_ versus *t*.

#### Intraparticle diffusion model

There are several phases involved in getting the adsorbate from the bulk liquid to the adsorbent surface. External mass transfer, intraparticle mass transfer, and pore surface adsorption are only a few of the steps that might have an impact on the total adsorption process. The potential of intraparticle mass transfer is examined using the intraparticle diffusion model. This model is expressed as in the following equation^38^:

15 $${q}_{t}={k}_{id}{t}^{0.5}+C$$

where; ${k}_{id}$ is the intraparticle diffusion rate constant (mg/g min^0.5^), $C$ is the constant of intraparticle diffusion. If a linear line obtained from drawing ${q}_{t}$ versus ${t}^{0.5}$, then the overall process of adsorption is specifically limited intraparticle mass transfer. However, if many linear plots emerged from graphing the data, then the adsorption process as a whole is strongly influenced by multiple phases of the previously listed stages^39^. The values of $C$ is the boundary layer thickness indication, the small value intercept means the weaker effect of the boundary layer^40^.

### Adsorbent's reuse

The regeneration was studied applying an exhausted MCM-48 adsorbent. Following the 4-nitroaniline adsorption solutions onto MCM-48, the mixture was filtered, the discarded adsorbent material that had uptake 4-nitroaniline was rinsed in a sizable amount of H_2_O until no 4-nitroaniline remained in solution, and then dried under vacuum conditions at 60 °C for the duration of an entire night. Several adsorption–desorption cycles were achieved in order to examine the MCM-48 material's resilience and potential for regeneration.

## Results and discussion

### Synthesized materials characterization

**Figure 1:**
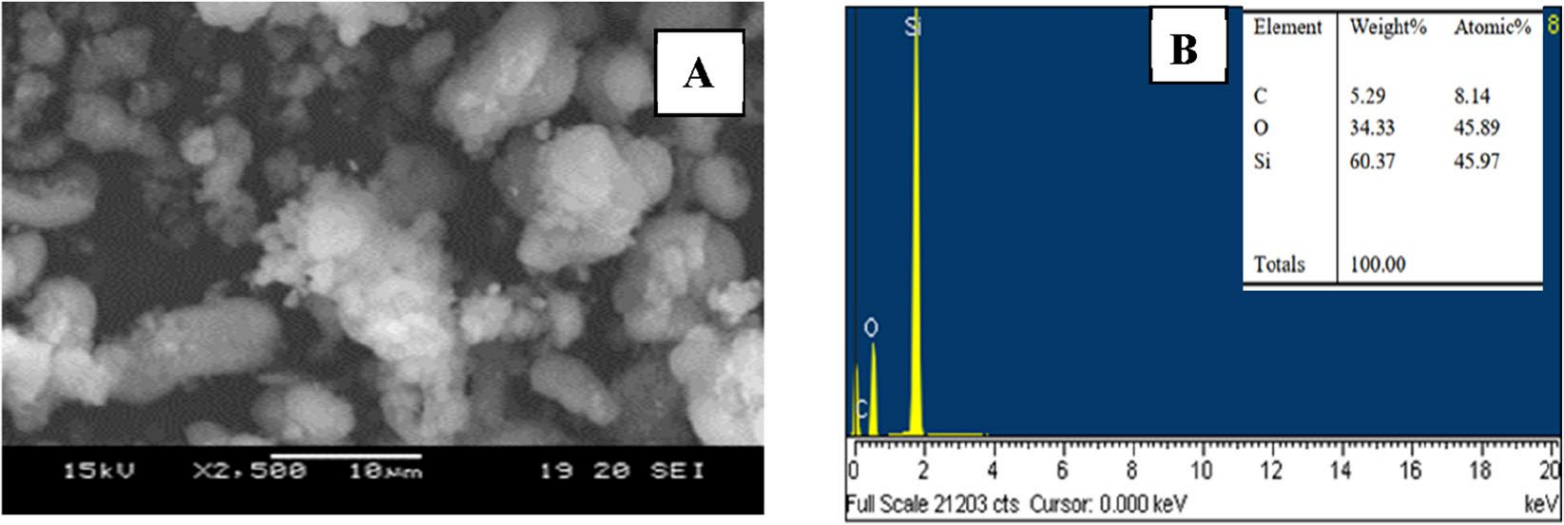
(**A**) SEM picture of MCM-48 at a 1000 × magnification. (**B**) A typical MCM-48 EDAX picture.

The prepared MCM- 48 was subjected to SEM and EDAX characterization procedures. At a magnification of 1000, SEM pictures of this material are displayed in Fig. 1A.

**Figure 2:**
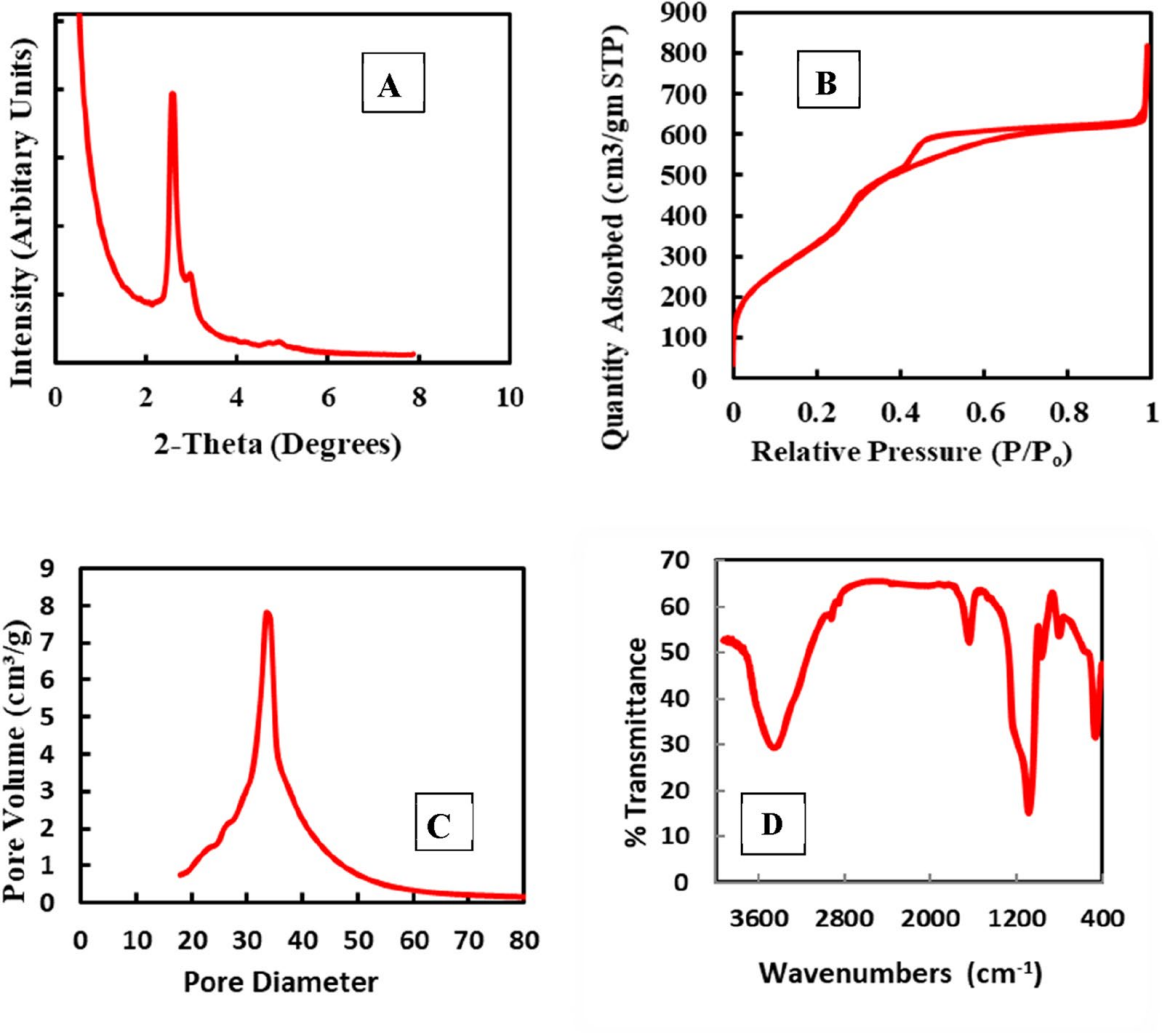
MCM-48 (**A**) X-ray diffraction pattern (**B**) isotherms of nitrogen adsorption–desorption, (**C**) BJH pore size distribution, and (**D**) FT-IR spectra.

The peaks on the EDAX graph in Fig. 1B show the components' average weight % values that C, O, and Si made up the zeolite. To get accurate average weight % for each of the components, EDAX graphs were created for a number of different locations on the SEM pictures. As can be displayed in Fig. 2A, the MCM-48 small angle XRD patterns exhibit a strong mesostructured-descriptive diffraction peak at around 2θ of 0.9°. Furthermore, two further peaks, indexed as (2 1 1), and (2 2 0) were seen in the XRD patterns. Two reflection peaks at 2θ less than 3° and a string of sporadic weak peaks in the range 3.5–5.5° are indexed to the Ia3d cubic structure. When compared to comparable peaks described in the literature, the peaks acquired in our investigation match those reported there quite well^41^. The outcomes shown in Table 1 illustrate how MCM-48 has a periodic ordered structure.

**Table 1:** MCM-48's physicochemical characteristics.

Sample	S_BET_ (m^2^/g)	V_P_ (cm^3^/g)	V_μP_ (cm^3^/g)	D_P_ (nm)	ɑ_o_ (nm)	t_wall_ (nm)
MCM-48	1420	1.2	0.4	3.7	3.4	0.6

According to Fig. 2B, the N_2_ adsorption isotherms of MCM-48 have a type IV isotherm and a type H1 hysteresis loop. A small pore size distribution is indicated by sharp adsorption and desorption branches. The sharpness and height of the capillary condensation process in the isotherms, in general, represent the uniformity of the pore size for mesoporous molecular sieves. In the relative pressure (P/P_0_) range of 0.05–0.25, the MCM-48 displays type IV isotherm, as illustrated in Fig. 2B. Together with the structural features discovered by nitrogen adsorption investigations, Table 1 explains the sample's specific surface area, pore size, pore volume, and wall thickness together with the structural characteristics obtained from nitrogen adsorption studies.

Figure 2C displays the pore size distribution (PSD) for the MCM-48. The content produced by CTAB: NaOH: The pore size distribution of TEOS is broad and centered at 35 Å. A mesopores higher amount was discovered for the basic synthesization, which created the most evident pore size dispersion and had a fairly regular organization^42^. Figure 2D displays the FT-IR spectrum of MCM-48, which includes the characteristic Si–O–Si bands at 1082, 964,799, and 460 cm^−1^. Stretching vibrations either Si OH or Si O Si can be attributed to the absorption band at around 960 cm^−1^. Because of the existence of the surface OH groups and the strong H_2_ bonding interactions between them, the wide band at approximately 3463 cm^−1^ is caused. Finally, the band at about 1637 cm^−1^ can be attributed to the distortion modes of the OH bonds of adsorbed H_2_O^43^.

### Adsorption of 4-nitroaniline

#### Effect of initial pH on the removal of 4-nitroaniline

**Figure 3:**
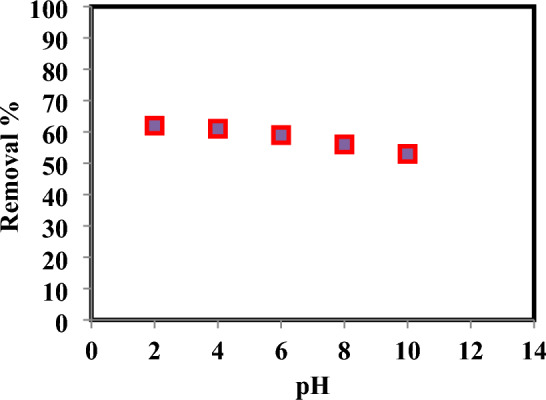
Effect of pH on removal of 4-Nitroaniline dye at contact time = 120 min, azo dy concentrations = 5 mg/L, temperature = 23 °C, adsorbents dose = 0.3 g, and shaking rotary speed = 200 rpm.

The pH of the bulk solution has a crucial role in the protonation and adsorption processes that remove 4-nitroaniline. The surface charge of the adsorbent, which in turn is regulated by the pH of the solution, is the main factor affecting the amount of absorption of dye ions onto the adsorbent surface^44^. As can be seen in Fig. 3, the removal efficiency of dye ions by the adsorbents dropped gradually as the solution's pH increased from pH 2.0 to pH 10.0, although it declined more sharply above pH 8.0. Due to the electrostatic repulsion effect, dye ions are less likely to be absorbed by adsorbents at low pH levels because protons compete with them for binding sites on the surface of the adsorbent. Higher pH levels result in a drop in the proton concentration, which increases the number of binding sites on the adsorbent and improves dye absorption.

#### Contact time effect

**Figure 4:**
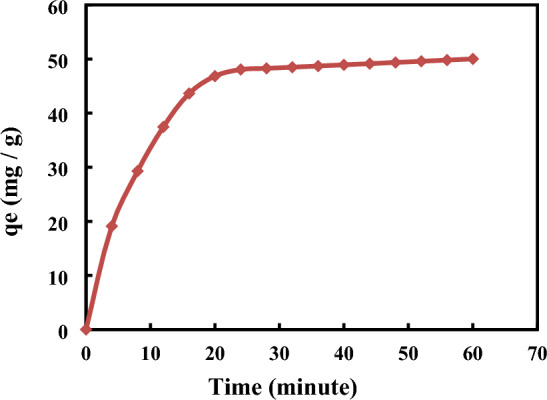
Contact time effect on the 4-Nitroaniline adsorption.

The contact time duration was determined for the 3-Nitroaniline solution to adsorb it onto MCM-48 in order to attain equilibrium as indicated in Fig. 4. The adsorption of 3-Nitroaniline solutions is clearly much influenced by time. The amount adsorbed on the nanoporous material zeolite was quantified for this purpose. The findings showed that equilibrium was established in under 20 min. It may take less time to attain balance. As a result, 60 min was specified to be the ideal contact time for the adsorbent. So, for the MCM-48 adsorbent to get saturated with analysis, just a very little contact time is needed. The adsorption capacity was greatly increased by higher cationic surfactant concentrations and their high availability in the pores of the adsorbent. This finding is significant, since one of the key factors taken into account for an efficient system of wastewater treatment is the equilibrium time. Therefore, in all experiments, adsorption was let to continue for 1 h^45^.

#### Agitation speed effect

The adsorption of hazardous solutes was examined by changing the agitation velocity speed from 0 to 200 rpm while maintaining a concentrated solution and contact time constant. The removal of harmful 4-nitroaniline solutions rose when the agitation velocity was raised from 0 to 150 rpm; however, it then remained constant. This suggests that an agitation velocity in the 150–200 rpm range is enough to ensure the surfactants optimum cationic sites present in the pores of MCM-48 adsorbent are rapidly made available for absorption. The best agitation speed for the remaining studies was determined to be 150 rpm^46^.

#### Initial concentration effect

**Figure 5:**
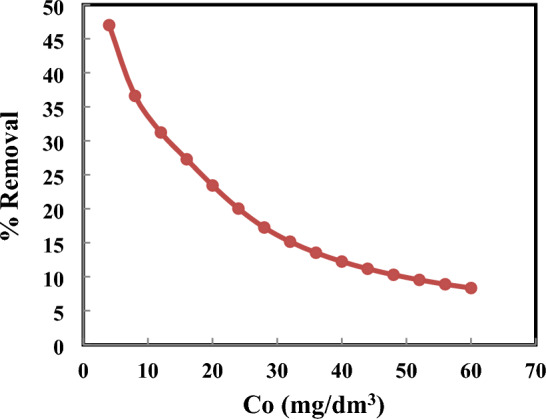
Initial 4-Nitroaniline concentration impact on removal efficiency at contact time = 60 min and MCM-48 dosage = 0.01 g.

The removal % of 4-nitroaniline was estimated from Eq. (1), as depicted in Fig. 5. Approximately 64% of the 4-nitroaniline content, which was initially 4 mg dm^−3^, is removed from solution. The removal % of 4-nitroaniline was decreased with the increased concentration at a constant mass of MCM-48. This reduces the amount of concentrated solution material that gradually absorbs more material. The quantity that may adsorb into the pores decreases when the maximum absorption of the MCM-48 pores is approached^47^.

### Adsorption isotherm

After testing for fitting to the various adsorption models, the best suited isotherm was established by taking into account the dynamic balance between the concentration of the adsorbate in the bulk solution and the concentration at the interface. The experiment revealed that the Langmuir adsorption is the best-fitting isotherm.

**Figure 6:**
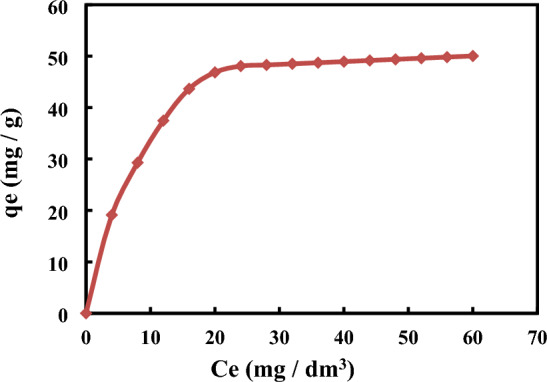
Isotherms of adsorption onto MCM-48 with 0.01 g/100 ml adsorbent.

Figure 6 depicts the 4-nitroaniline compound's adsorption isotherms, where Ce represents the adsorbate equilibrium concentration in solution at equilibrium and qe represents the adsorbate adsorbed per gram of MCM-48. 4-nitroaniline molecule was generally adsorbed throughout a variety of concentrations, demonstrating the efficiency of MCM-48 in the removal of 4-nitroaniline from aqueous solutions as an adsorbent. An essential driving factor for overcoming all ion and molecule mass transfer resistances between the solid phases and aqueous is the initial adsorbate concentration^48^. The initial solution concentration in the current investigation is adjusted from 4 to 60 mg/L however, the adsorbent dose stays constant at 0.01 g/100 mL. Figure 6 shows that when the equilibrium adsorption capacity increases, the initial 4-nitroaniline concentration also rises. The uptake of equilibrium adsorption for 4-nitroaniline rose from 20 to 89 mg/g as the starting concentration solution of 4-nitroaniline was increased from 4 to 60 mg/L. This is because of the excess in number of 4-nitroaniline molecules of poisonous solutes vying for the few remaining sites of binding onto the adsorbent's surface. According to the finding shown in Fig. 6, cationic surfactants that include MCM-48 have a very high uptake for 4-nitroaniline adsorption. As seen in Fig. 2D, silanol groups (Si–OH) constitute significant sites of adsorption on the MCM-48surface. These sites are present in this substance in addition to cationic sites that were provided by a cationic template. It appears that a key determinant in the adsorption of 4-nitroaniline on the surface of MCM-48 is the number of sorption sites. This can be explained by the fact that there are initially lots of empty surface sites accessible for adsorption. It was also proposed that as time went on, a potent attraction force developed between the molecules of 4-nitroaniline and the sorbent. Due to saturation, it is difficult to fill the remaining open surface sites, which may also be related to a lack of accessible sorption sites towards the conclusion of the adsorption process^49^.

#### Langmuir isotherm model

**Figure 7:**
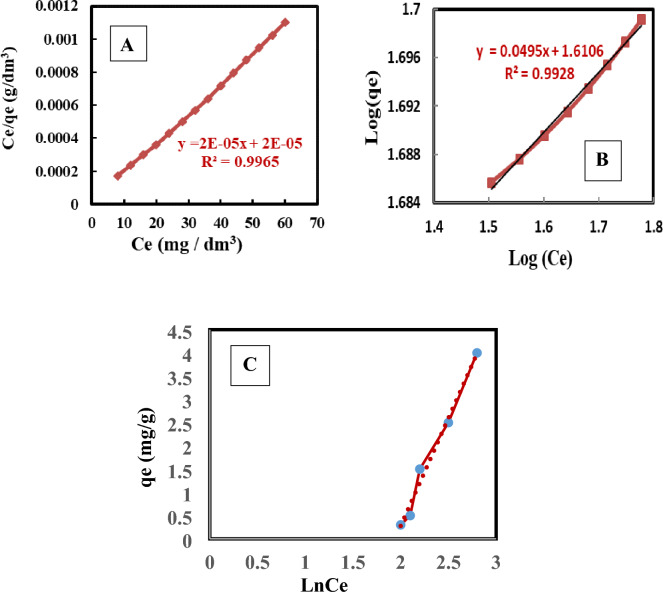
4-Nitroaniline adsorption onto MCM-48 according to (**A**) Langmuir, (**B**) Freundlich, and (**C**) Temkin isotherms.

The adsorption isotherms for 4-nitroaniline compound have profiles that are consistent with Type I Langmuir adsorption; the quantity adsorbed rose gradually until it reached values in the 25–89 mg g^−1^ range. The study of adsorption isotherms has been done so as to model the adsorption behavior. According to Eq. (5), the Langmuir isotherm models was applied to assess the 4-nitroaniline species adsorption process, as shown in Fig. 7A. Graphing the Eq. (5) linear form of, or **Ce/qe** versus **Ce**, which was applied to determine the constants of Langmuir and maximal uptake or capacity qmax, corroborated type I adsorption. According to Table 2 and Fig. 7A, the Langmuir profile for the 4-nitroaniline molecule is a straight line, supporting Type I adsorption. **R**^**2**^ values are 0.99. Figure 7 displays the linearized isotherm data using the Langmuir equation. Table 2 lists the regression coefficients. R^2^ = 0.9965, a high correlation coefficient, denotes strong agreement between the parameters. 4-nitroaniline has an adsorption ability to create monolayers that may reach up to 90 mg/g, according to the constant qmax. The value of the adsorption energy constant, b, for 4-nitroaniline is 1 dm^3^/mg. The Langmuir equation can also be expressed in terms of a dimensionless separation factor or an equilibrium parameter R_L_, which can be used to predict the favorability of an adsorption system. Table 2 shows the RL values based on the Langmuir isotherm model, with all R_L_ values greater than 0 but less than 1 indicating that the Langmuir isotherm is favorable^50,51^.

**Table 2:** Constant of Langmuir, Freundlich, and Temkin for 4-Nitroaniline adsorption on MCM-48.

Adsorbate	Langmuir Constants					Freundlich constants		Temkin constants		
	qmax (mg g^−1^)	b (dm^3^ mg^−1^)	R^2^	K_F_	R_L_	1/n	R^2^	b_T_	Ln K_T_	R^2^
4-Nitroaniline	90	1	0.9965	40.794	0.16	0.0495	0.9929	537.69	−1.95	0.9834

#### Freundlich isotherm model

According to Eq. (8), Freundlich isotherm models was used to assess the 4-nitroaniline species adsorption process. The Freundlich equation was also fitted to the similar data, which is seen in Fig. 7B. Table 2 provides the constants of regression. The correlation coefficient values demonstrate how closely the data follow the Langmuir equation. The 4-nitroaniline component adsorption on mesoporous materials is better depicted by the Langmuir model than by the Freundlich model. Furthermore, 4-nitroaniline has 1/n values that are smaller than 1, which is a sign of a high adsorption intensity^52^.

#### Temkin isotherm model

Temkin's adsorption model takes into account how the adsorbate and adsorbing species interact, according to Eq. 10^35^. The model predicts that at relatively low concentrations, the interaction between adsorbate and adsorbent will cause the heat of adsorption, which is a function of temperature, to fall linearly rather than logarithmically with degree of coverage for all the molecules in the layer. A plot of ${q}_{e}$ versus $\mathrm{ln}$ ${\mathrm{C}}_{\mathrm{e}}$ enables the determination of the isotherm constants ${K}_{T}$ and ${b}_{T}$.

Where; ${b}_{T}$ is the constant of Temkin, which is connected to adsorption heat (kJ/mol), and ${K}_{T}$ is the actual Temkin constant connected to the constant of equilibrium binding and the maximum binding energy (L/mg). The adsorption of 4-nitroaniline dye on the surface of MCM-48 according to Temkin isotherm is shown in Fig. 7C, while adsorption parameters deduced from the plots are recorded in Table 2.

### Adsorption kinetics

One of the most important elements that defines the effectiveness of adsorption is the rate of 4-nitroaniline adsorption by MCM-48. A pseudo-first and second order model have been applied to explain kinetics of 4-nitroaniline adsorption. Figures 7 (A) and (B) illustrate how pseudo-first- and second-order kinetics models were used to analyze the 4-nitroaniline kinetics of adsorption onto MCM-48. Table 3 contains the results for the kinetic model parameters and the correlation coefficients (R^2^)^53^.

**Table 3:** Kinetics parameters values for 3-Nitroaniline adsorption on MCM-48.

Adsorbates	qe.exp (mg/g)	Pseudo-first order constants			Pseudo-second order constants			Intraparticle diffusion constants	
		qe.cal (mg/g)	K_1_ (g/mg min)	R^2^	qe.cal (mg/g)	K_2_ (g/mg.min)	R^2^	K_id_ (mg/g min^0.5^)	R^2^
4-Nitroaniline	50	107	0.019	0.979	163	1.042*10^–4^	0.994	2.893	0.953

#### Pseudo 1st and 2nd order kinetic model

**Figure 8:**
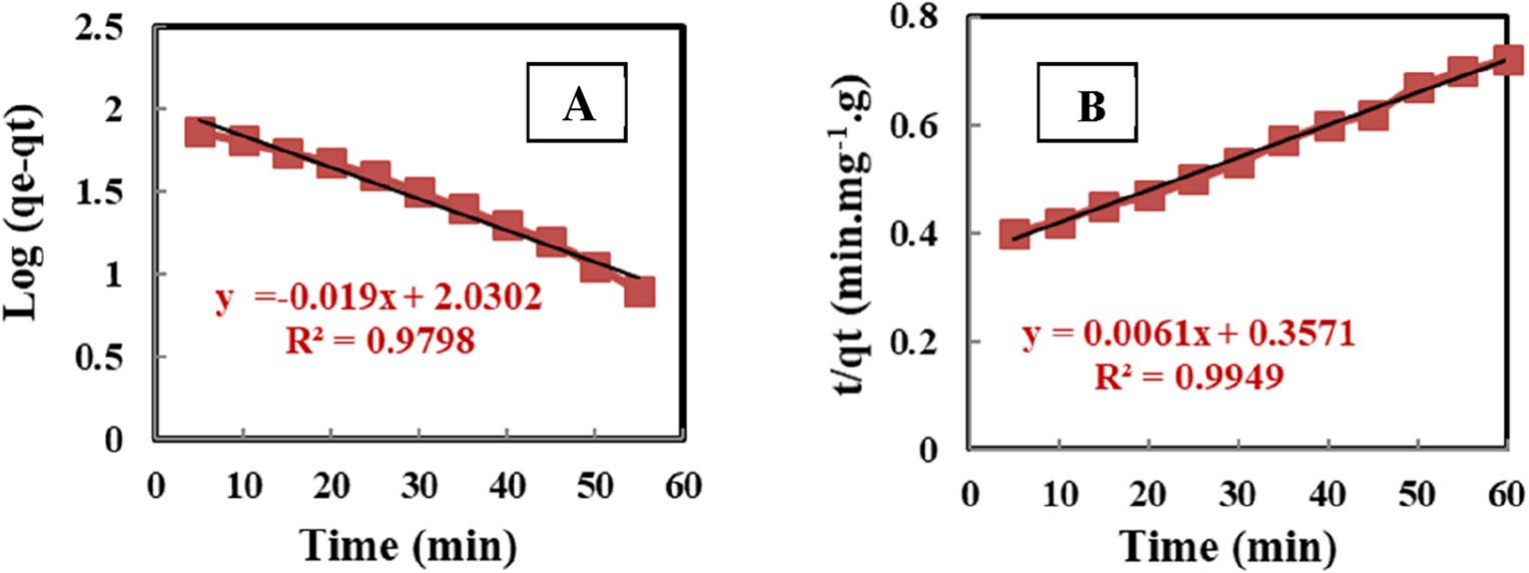
(**A**) Pseudo 1st and (**B**) 2nd order kinetics for the 4-Nitroaniline adsorption on MCM-48.

According to Table 3, the theoretical values (qe cal.) calculated from the pseudo-first-order kinetic model, as presented in Eq. (12). It is giving substantially different values when compared to experimental values (qe exp.). As a result, the pseudo 1^st^ order kinetic model is representing this adsorption system well. The theoretical (qe cal.) and the experimental values (qe exp.) were calculated using the pseudo 2nd order kinetic model, as shown in Eq. (14). The values are extremely close to each other, as shown in Table 3. The coefficients R^2^ value is also quite near to 1, which supports the validity of the pseudo-second-order equation. In the current research, the adsorption data were analyzed applying two important kinetic models^54^. Figure 8A, B explain the findings of Pseudo-first and second order models were used to fit the experimental data, respectively. The results from the adsorption kinetics are extremely well matched by the pseudo-second order model, as shown in Fig. 8B. Given that 4-nitroaniline regression coefficients are so high R^2^ = 0.9949, the pseudo 2nd order hypothesis for the adsorption mechanism process appears to be well-supported ^22^.

#### Intraparticle diffusion model

**Figure 9:**
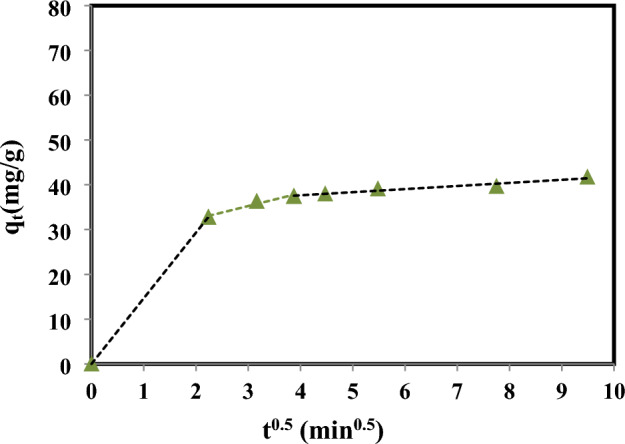
Intraparticle diffusion of 4-Nitroaniline adsorption onto MCM-48.

The experimental results were applied to the intraparticle diffusion model according to Eq. (15), in order to learn more about the mechanism and rate-controlling processes, as shown in Fig. 9. The figure that displays multi-linear plots represents the rate of change in 4-Nitoanile adsorbed in three phases, as shown by the dotted line. Due to the availability of free adsorption sites on the outside of MCM-48 and the greater concentration gradient of 4-nitroaniline ions, the adsorption rate in the first stage increases rapidly. In this stage and according to Fig. 9, which shows dependence on external diffusion, the higher gradient of concentration is shown by the bigger slope of the dotted lines in this region. After this stage and over time, the amount of 4-nitroaniline adsorbed considerably decreased. This was caused by the saturation of MCM-48's external sites and a decline in the gradient of 4-nitroaniline concentration. The rate of adsorption in this region is slower than in the first stage because 4-nitroaniline ions will be transported from the bulk of the solution to the adsorbent external surface and subsequently diffuse within pores. Because the second section of the plots in the picture does not have a zero intercept, intraparticle diffusion is not the sole rate that affects the adsorption process. Table 3 displays the results of calculating the values of ${k}_{id}$ obtained from this step using the slope of the second stage line. Table 3 shows that ${k}_{id}$ values is 2.8938 mg/g min^0.5^ with determination coefficient R^2^ = 0.9537. The horizontal dotted line of Fig. 9 appears very slower uptake of 4-nitroaniline ions occurs in the third region because of the equilibrium adsorption^55^.

### Adsorption mechanism

**Figure 10:**
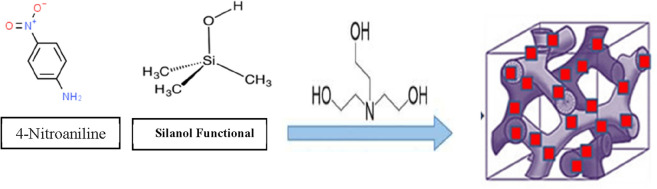
Mechanism adsorption of 3-Nitroaniline onto MCM-48 adsorbent.

To understand the adsorption mechanism process, it is important to know the structure of the adsorbent and adsorbate as shown in Fig. 10. 4-nitroaniline molecule is an organic chemical substance, particularly a primary aromatic amine. It consists of an amino group attached to a benzene ring. On the other hand, MCM-48 adsorbent is the most common molecular sieves of mesoporous materials that is intensively investigated by researchers. The most notable feature of the MCM-48 is that despite having an amorphous silica wall, it has a long-range organized structure and consistent mesoporous. This important material has the majority of silanol group. Based on the structure of the 4-nitroaniline, MCM-48 and experimental results of kinetic, FTIR and EDX analysis, the adsorption mechanism of 4-nitroaniline onto MCM-48 adsorbent can be determined. Based on FTIR results, the strong adsorption band of at –C=C– group was decreased in intensity and shifted from 1681 to 1600 cm^−1^ owing to π–π interactions between 4-nitroaniline molecule with –C=C–onto the surface of MCM-48. While the band of –OH groups was increased in intensity and a slight shift from 3330 to 3335 owing to hydrogen bond formation between the –N(CH_3_)_2_ group of 3-Nitroaniline molecules and the –OH group in the MCM-48 surface. The band of C=O stretch vibration of carboxylic acid was a slight increase in intensity due to electrostatic attraction between a cationic ^+^N(CH_3_)_3_^+^ group of MG molecules with the negative charge of COOH group onto the MCM-48 surface. According to the results of adsorption isotherms and kinetics study, the adsorption mechanism is a chemisorption and physical adsorption process. Therefore, these results provide enough evidence to support the 4-nitroaniline adsorption onto the MCM-48 surface by different mechanisms such as hydrogen bonding, electrostatic interaction and π–π interactions^56,57^.

### Adsorbent reuse

The possibility of recycling the adsorbent was examined for applying a depleted MCM-48 after regeneration. For the purpose of proving that the MCM-48 could be regenerate by removing the adsorbates, desorption investigations were carried out. When using the MCM-48 again, it is crucial to establish that desorption takes place. According to the results of the trials, 4-nitroaniline was effectively and efficiently desorbed into deionized water in a single cycle, with an efficiency of over 90%. It can be seen from this that all used materials may be recycled. It is possible to perform a more thorough investigation to learn more about desorption, including the impacts of solution concentration, adsorbate loading, temperature, etc. The focus of this essay does not extend to this^58^.

### Comparative study

Table 4 compares the MCM-48 with several other adsorbents mentioned in the literature. This table offers important details regarding how effectively MCM-48 adsorbent may increase 4-nitroaniline's capacity for adsorption from other adsorbent materials. Pure MCM-48 has an adsorption capability of 90 mg g^−1^ for 4-nitroaniline without functionalization or treatment of its surface. One may conclude that adding a functional group to the surface of MCM-48 will increase its adsorption capacity. Table 4 shows that MCM-48 is a superior adsorbent for removing 4-nitroaniline dye because it has a higher surface area than the other adsorbents, reaching 1420 m^2^/g.

**Table 4:** Adsorption capacities of 4-Nitroaniline by various adsorbents.

No.	Adsorbents	Adsorption capacity Q_max_ (mg g^−1^)	References
1	Modified γ-Aluminum oxide (γ-Al_2_O_3_-Silane-Cl)	97.97	^59^
2	Polymeric adsorbent HJ-02	90.25	^60^
3	Magnetic biochar (MDFBC)	114	^61^
4	Spartina alterniflora activated carbon (SAAC)	719	^62^
5	Multiple functional nanocomposite (GQDs@Gem@FA-DLH)	65.6	^63^
6	Mesoporous materials MCM-48	90	This study

## Conclusion

In this study, 4-nitroaniline was easily adsorbed from an aqueous adsorbate solution utilizing from the MCM-48 mesoporous material. According to a linear analysis with R^2^ values of 0.99, 4-nitroaniline molecules matched to Type I Langmuir adsorption. Under appropriate experimental circumstances, nanoporous material demonstrated considerable aniline adsorption capacity; as a result, it may be suggested as a practical adsorbent. The Langmuir adsorption isotherm was discovered the best suited with the experimental, indicating monolayer adsorption on a homogeneous surface. The Langmuir isotherm was used to calculate the maximal adsorption capacity of mesoporous adsorbent and it was found 90 mg g^−1^. The pseudo-second-order model can forecast the dynamics of adsorption. Adsorption isotherms and kinetics models indicate that chemical and physical adsorption are the two processes involved in the adsorption mechanism.

## Data Availability

All relevant data and material are presented in the main paper.
